# Transient atrial inflammation in a murine model of Coxsackievirus B3‐induced myocarditis

**DOI:** 10.1111/iep.12438

**Published:** 2022-04-01

**Authors:** Linghe Wu, Mitchell D. Fiet, Daan R. Raaijmakers, Linde Woudstra, Albert C. van Rossum, Hans W. M. Niessen, Paul A. J. Krijnen

**Affiliations:** ^1^ Department of Pathology Amsterdam University Medical Centers, location VUmc Amsterdam The Netherlands; ^2^ Department of Pathology Amsterdam University Medical Centers, location AMC Amsterdam The Netherlands; ^3^ Amsterdam Cardiovascular Sciences Amsterdam The Netherlands; ^4^ Department of Reproductive Medicine Reinier de Graaf Hospital Voorburg The Netherlands; ^5^ Department of Cardiac Surgery Amsterdam UMC, location VUmc Amsterdam The Netherlands; ^6^ Department of Cardiology Amsterdam UMC, location VUmc Amsterdam The Netherlands

**Keywords:** atria, Coxsackievirus B3, in situ hybridization, inflammation, viral myocarditis

## Abstract

Atrial dysfunction is a relatively common complication of acute myocarditis, although its pathophysiology is unclear. There is limited information on myocarditis‐associated histological changes in the atria and how they develop in time. The aim of this study therefore was to investigate inflammation, fibrosis and viral genome in the atria in time after mild CVB3‐induced viral myocarditis (VM) in mice. C3H mice (*n* = 68) were infected with 10^5^ PFU of Coxsackievirus B3 (CVB3) and were compared with uninfected mice (*n* = 10). Atrial tissue was obtained at days 4, 7, 10, 21, 35 or 49 post‐infection. Cellular infiltration of CD45+ lymphocytes, MAC3+ macrophages, Ly6G+ neutrophils and mast cells was quantified by (immuno)histochemical staining. The CVB3 RNA was determined by in situ hybridization, and fibrosis was evaluated by elastic van Gieson (EvG) staining. In the atria of VM mice, the numbers of lymphocytes on days 4 and 7 (*p* < .05) and days 10 (*p* < .01); macrophages on days 7 (*p* < .01) and 10 (*p* < .05); neutrophils on days 4 (*p* < .05); and mast cells on days 4 and 7 (*p* < .05) increased significantly compared with control mice and decreased thereafter to basal levels. No cardiomyocyte death was observed, and the CVB3 genome was detected in only one infected mouse on Day 4 post‐infection. No significant changes in the amount of atrial fibrosis were found between VM and control mice. A temporary increase in inflammation is induced in the atria in the acute phase of CVB3‐induced mild VM, which may facilitate the development of atrial arrhythmia and contractile dysfunction.

## 1 INTRODUCTION

Myocarditis is an inflammatory disease of the heart that most often has a viral aetiology. Myocarditis can lead to acute and chronic heart failure and sudden death, but may also present with only mild symptoms such as fever and fatigue and is believed to most often occur asymptomatically.^1^ Moreover, arrhythmia of the atria is a relatively common complication of myocarditis, also of acute myocarditis.^2^, ^3^, ^4^ However, the pathophysiology of atrial dysfunction in myocarditis is incompletely understood.

Histopathologic analyses of atrial tissue obtained from patients with atrial fibrillation have revealed inflammatory cell infiltration and an increase in fibrosis,^5^, ^6^ which are suggested to contribute to proarrhythmogenic remodelling of the atria. Several studies have shown that in myocarditis, the atria are also affected. In a BALB/c mouse model of acute Coxsackievirus B3 (CVB3)‐induced myocarditis, inflammatory cell infiltration and mild‐to‐moderate cardiomyocyte necrosis were observed up to 21 days after infection, although changes in fibrosis were not reported.^7^ This was accompanied by a viral infection of the atrial myocardium, which was similar in kinetic and extent to the ventricular myocardium.^7^ In addition, we have previously observed a similar increase in inflammatory cell infiltration in the atria of patients with lymphocytic myocarditis (LM).^8^ However, we did not observe cardiomyocyte death in the atria nor an increase in fibrosis, while the putative viral presence in the atria was not analysed. The differences in the observed histopathological changes in the atria in these studies may relate to differences in disease severity between the mice and humans. The majority of patients with myocarditis have a mild or asymptomatic course, and autopsy findings reveal that in most LM patients, the extent of the inflammation and cardiomyocyte necrosis in the ventricles are quite limited.^7^, ^9^, ^10^ Recently, we have shown that the ventricular inflammatory infiltrates can form an important arrhythmogenic substrate in C3H mice wherein CVB3 infection resulted in mild acute myocarditis, with relatively little ventricular cardiac damage.^11^

The aim of this study therefore was to examine the extent of inflammation, fibrosis and viral genome in the atria in time after mild CVB3‐induced viral myocarditis (VM) in mice.

## 2 METHODS AND MATERIALS

### 2.1 Mouse model

Seventy‐eight male C3H mice (Harlan) were divided into uninfected control mice (control group, *n* = 10) and CVB3‐infected VM mice (VM group, *n* = 68). The CVB3 groups received an intraperitoneal (i.p.) injection containing 10^5^ plaque‐forming units (PFU) of enterovirus Coxsackievirus B3 (CVB3: Nancy strain; ATCC) on Day 0,^11^ while the control group received an i.p. saline injection and were sacrificed at Day 14 (*n* = 10) post‐injection. No mice died prematurely during the experiments, and all mice survived after CVB3 injection until sacrifice. VM groups were sacrificed at Day 4 (*n* = 10), Day 7 (*n* = 10), Day 10 (*n* = 9), Day 14 (*n* = 10), Day 21 (*n* = 10), Day 35 (*n* = 10) and Day 49 (*n* = 9) post‐viral infection. All left and right atrial tissue were excised and fixed in 4% formaldehyde and embedded in paraffin. The tissue from these mice was used in a previous study.^11^ This study was approved by the Animal Ethics Committee of the VU University (Amsterdam, the Netherlands) and was in accordance with ethical guidelines published by the European Commission Directive 2010/63/EU.

### 2.2 (Immuno)histochemical analysis

Serial cross sections (4 µm) of the atria and ventricles (for fibrosis determination only) of VM and control mice were stained with antibodies detecting CD45 (lymphocytes), CD3 (T lymphocytes), MAC3 (macrophages) and Ly6G (neutrophils). A histological toluidine blue was used to identify mast cells. The sections were first deparaffinized, rehydrated and blocked for endogenous peroxidases by incubation in methanol with 0.3% H_2_O_2_ for 30 min. The sections were then stained with haematoxylin–eosin (HE) and with elastic van Gieson (EvG) to detect fibrosis. As an antigen retrieval step, the slides were heated in a 0.01 M citrate buffer (pH = 6.0; for CD45 and MAC3 stainings) or in a 10 mM Tris‐EDTA buffer (pH 9.0; for CD3 staining) for 15 min or incubated with activated pepsin at 37°C for 30 min (for Ly6G staining). The slides were incubated in Normal Rabbit Serum (1:100, Dako X0902; CD45, MAC3, Ly6G) or Normal Swine Serum (1:20, X10964; Monosan) for 10 min at room temperature (RT). Thereafter, the sections were subsequently incubated with either rat anti‐mouse CD45 antibody (1:50 dilution; BD Pharmingen, clone 30‐F11, cat. no. 553076), rabbit anti‐mouse CD3 (1:100 dilution; Abcam, clone SP7, cat. no. Ab16669), rat anti‐mouse MAC3 antibody, (1:30 dilution; BD Pharmingen, clone M3/84, cat. no. 553322) or rat anti‐mouse Ly6G antibody (1:200 dilution; Abcam, cat. no. 4066611) for 60 min at room temperature. The slides were then washed with phosphate‐buffered saline and incubated with Real EnVision HRP α‐mouse/rabbit (undiluted; Dako, cat. no. K5007) (for CD45 and MAC3 stainings), α‐rabbit (undiluted; Dako, cat. no. K4003) (for CD3 staining) or mouse anti‐rat biotin‐labelled antibody (1:100; Jackson ImmunoResearch, cat. no. 307329) (for Ly6G staining) for 30 min at room temperature. The Ly6G‐stained slides were then incubated with streptavidin–HRP (1:100; Dako, cat. no. P0937) for 60 min and visualized with AEC (3‐amino‐9‐ethylcarbazole; Invitrogen, cat. no. 911667A), and the CD45 and MAC3 stainings were visualized using 3,3‐diaminobenzidine (0.1 mg/ml, 0.02% H_2_O_2_). All slides were counterstained with haematoxylin, dehydrated and covered. A negative control (without primary antibody) was included in each staining, and all these controls showed no staining (data not shown).

### 2.3 Quantification of inflammatory cells

The numbers of extravasated CD45+ cells (lymphocytes), CD3 (T lymphocytes), MAC3+ cells (macrophages) and mast cells in the atria were quantified blinded using a light microscope at 500× magnification (Zeiss). The slides were then scanned using a PathScan Enabler IV scanner (Meyer Instruments). The total surface area of the analysed tissue was determined on these scans using the QuickPhoto Microanalysis software (Promicra). In addition, this software was used to determine the mean surface area of the lesions and fibrosis. The number of inflammatory cells per mm^2^ was calculated as the total score for each specimen. The presence of neutrophils was quantified as the percentage of the Ly6G‐positive area of the tissue using the ImageJ software. Immunoscoring was performed by L. Wu, PAJ. Krijnen and HWM Niessen. An agreement was reached between these observers.

### 2.4 In situ hybridization

To detect CVB3 RNA in the atrial tissue, RNA‐ISH using the RNAscope 2,5 HD Detection Kit (Advanced Cell Diagnostics (ACD); cat. no. 322310) was performed according to the manufacturer's instructions. The slide‐mounted 4‐μm paraffin tissue sections were warmed at 60°C for 1 h, followed by a 5‐min incubation in xylene and 100% ethanol. The slides were then successively incubated in RNAscope^®^ Hydrogen Peroxide for 10 min at room temperature, in target retrieval solution (ACD; cat. no. 322000) for 15 min at 99°C and finally in RNAscope^®^ Protease Plus solution for 30 min at 40°C to allow probe access with intermittent rinses in distilled H_2_O. For the hybridization step, the slides were incubated with the CVB3 probe (ACD; cat. no. 409291) for 2 h at 40°C. For signal amplification, the slides were successively incubated with AMP1 (30 min at 40°C), AMP2 (15 min at 40°C), AMP3 (30 min at 40°C), AMP4 (15 min at 40°C), AMP5‐Brown (30 min at room temperature; HRP‐labelled) and AMP6‐Brown (15 min at room temperature; HRP‐labelled) with intermittent washes in wash buffer. The slides were then incubated with DAB (10 min at room temperature) for signal detection, counterstained in haematoxylin, dehydrated and covered. For each test, a positive and negative control probe was included to confirm tissue RNA quality and determine a specific background staining. The slides were analysed using light microscopy.

### 2.5 Statistical analysis

Statistical analyses were performed with the spss statistics program (Windows version 21.0). Statistical significance between groups was analysed with a Student t test for normally distributed data or with a Mann–Whitney test for non‐normally distributed data. The comparisons between multiple groups (more than two) were evaluated by either a one‐way ANOVA or a Kruskal–Wallis test with post hoc Dunn's multiple comparisons for normal or non‐normal distributed data. Correlations were determined using Pearson's *r* or Spearman's rank correlation coefficient depending on the distribution of the data. A correlation coefficient ranging from 0.50 to 1.00 or −0.50 to −1.00 indicates a strong correlation. We also calculated the coefficient of determination (CoD) for the correlation. A *p*‐value <0.05 was considered significant.

## 3 RESULTS

### 3.1 Transiently increased inflammatory cell infiltration in the atria in time after VM

**FIGURE 1:**
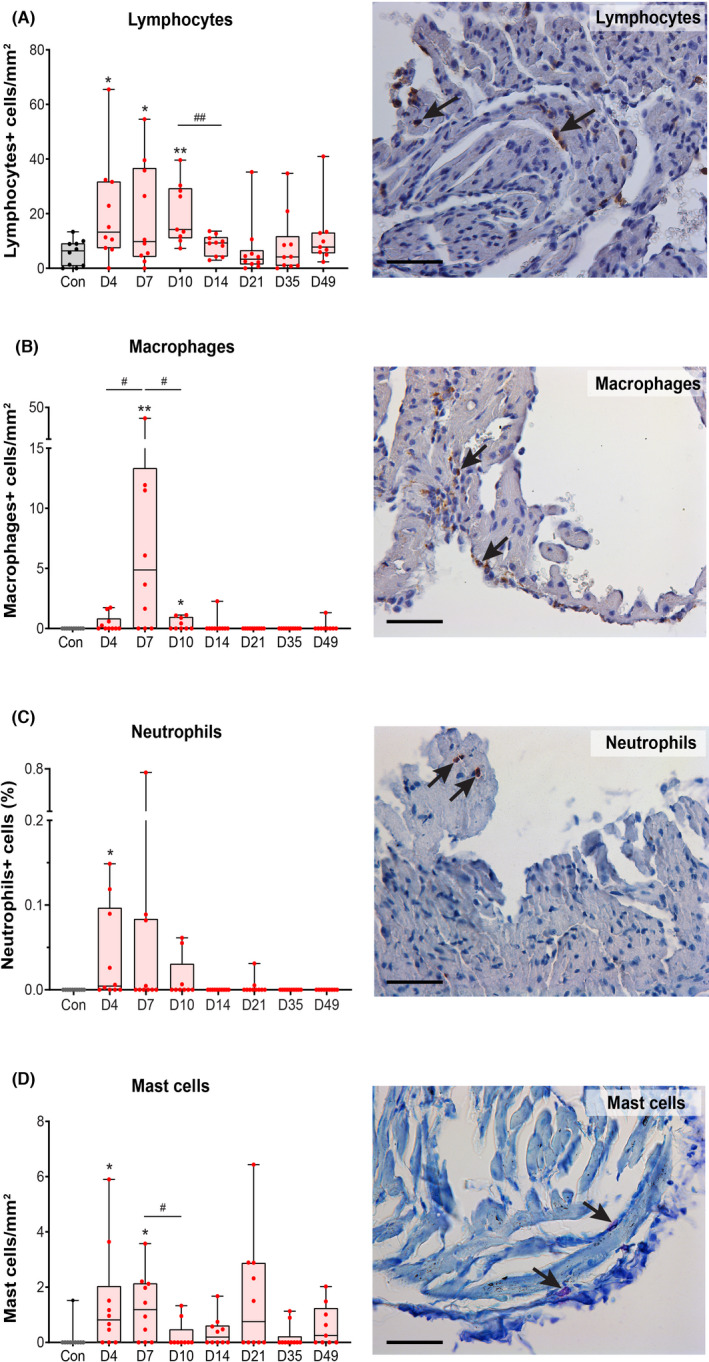
Quantification of inflammatory cells in the atria in control (con, *n* = 10) and viral myocarditis (VM) mice (total *n* = 68) at different time points after CVB3 infection (D = day post‐infection). (A) CD45‐positive cells (lymphocytes). (B) MAC3‐positive cells (macrophages). (C) Ly6G‐positive cells (neutrophilic granulocytes). (D) Mast cells. A‐D: The arrows in the pictures depict the respective inflammatory cells. A/B and D are shown as the number of cells per mm^2^ in the heart. C is depicted as the surface area of Ly6G staining relative to the total ventricular surface area in the heart, Scale bar = 50 µm. Data are medians and interquartile ranges. *means compared with the control group, *p < .05, **p < .01, ^#^ *p* < .05 and ^##^ *p* < .01

The numbers of CD45+ cells in the atria were significantly increased on days 4 (*p* < .05), 7 (*p* < .05) and 10 (*p* < .01) post‐infection compared with the control group (Figure 1A). On Day 14, the number of CD45+ cells decreased significantly compared with that on Day 10 (*p* < .01) and remained low thereafter until Day 49. Notably, we observed relatively low numbers of CD3+ cells (0.5–6 cells/mm^2^) present in the atria of only part of the VM mice at all time points post‐infection, while none were found in the control mice.

Significant increases in macrophages were observed later, on days 7 (*p* < .01) and 10 (*p* < .05), but returned to control levels thereafter (Figure 1B). A clear peak in atrial macrophages was observed on Day 7, where the numbers were significantly higher than on days 4 (*p* < .05) and 10 (*p* < .05). A significant increase in neutrophils was found only on Day 4 (*p* < .05, Figure 1C), and minor but significant increases in mast cells were found on days 4 and 7 post‐infection (*p* < .05; Figure 1D). No cell damage or microcalcifications were observed in the atrial tissue in any of the groups. A correlation analysis revealed only a strong positive correlation between the numbers of macrophages and mast cells on days 7 (*r* = .530, CoD = 0.281, *p* = .115), 14 (*r* = .557, CoD = 0.715, *p* = .1) and 49 (*r* = .709, CoD = 0.503, *p* = .033). In conclusion, a transient increase in infiltrated lymphocytes, macrophages, neutrophils and mast cells, without cardiomyocyte death, was observed in the atria during the CVB3‐induced VM progression, especially on days 4 and 7 post‐infection.

### 3.2 Marginal presence of Coxsackievirus B3 in the atria of VM mice

**FIGURE 2:**
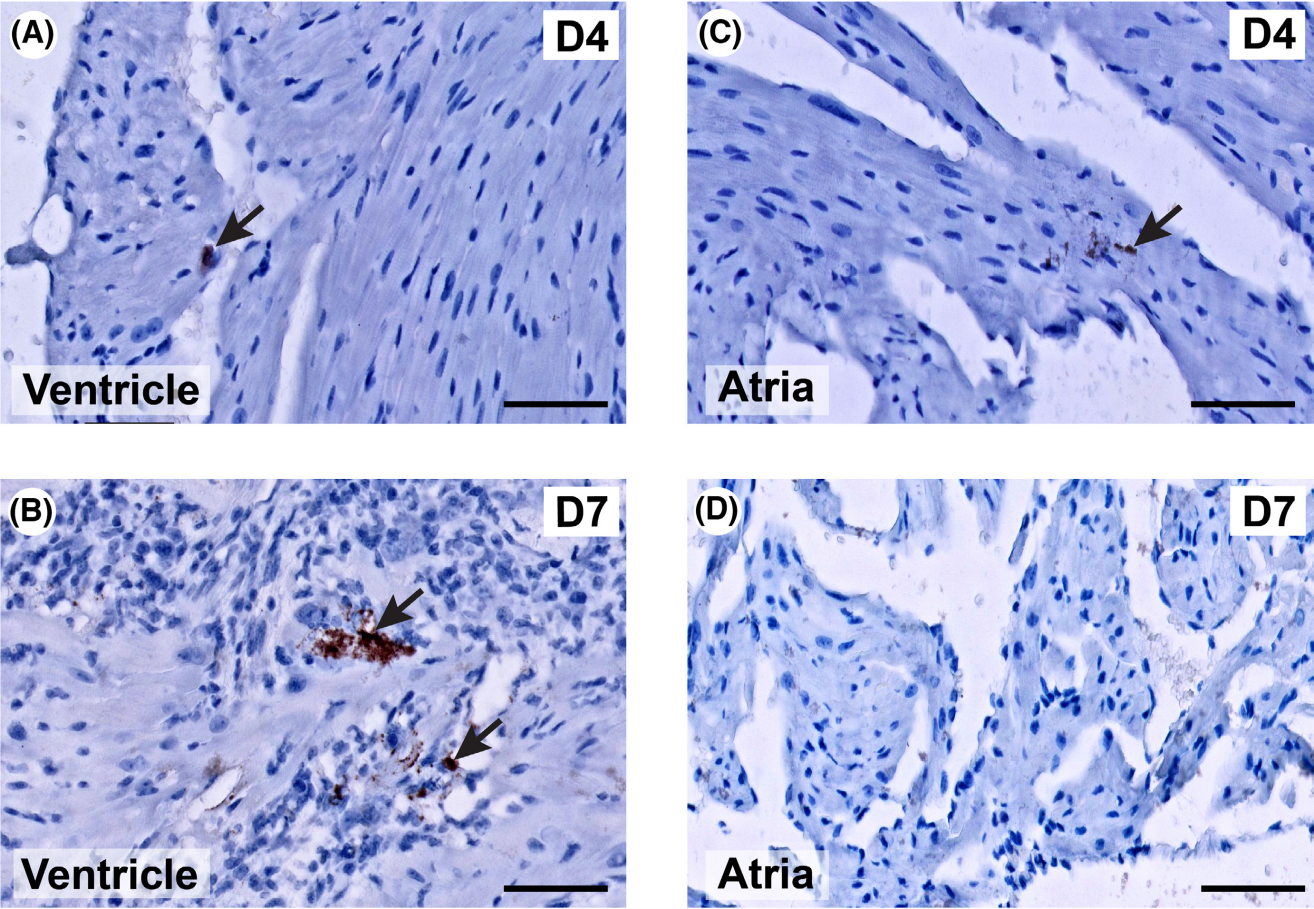
In situ hybridization detection of CVB3 RNA in the hearts of viral myocarditis (VM) mice during CVB3 infection. Shown are examples of ventricular (A and B) and atrial (C and D) tissues obtained at Day 4 (D4) and Day 7 (D7) post‐infection. The arrows depict CVB3 RNA (granular brown staining). Scale bar = 50 µm

In situ hybridization (ISH) was performed to investigate the presence of the Coxsackie B3 virus (Figure 2). Ventricular tissue was used as the positive control. In the ventricular myocardium, the CVB3 genome was found to be localized to the cytoplasm of jeopardized cardiomyocytes and the interstitial space in areas of inflammation, 4 (Figure 2A) and 7 days (Figure 2B) post‐infection. In the atria, very limited CVB3 RNA was detected in the atrial tissue on Day 4 post‐infection in only 1 mouse (Figure 2C), whereas no viral RNA was present on Day 7 (Figure 2D) nor at a later time point. No ISH signal was detected in control mice. These results show only a marginal presence of virus in the atria of VM on Day 4 post‐infection.

### 3.3 No significant differences in atrial fibrosis in time after VM

**FIGURE 3:**
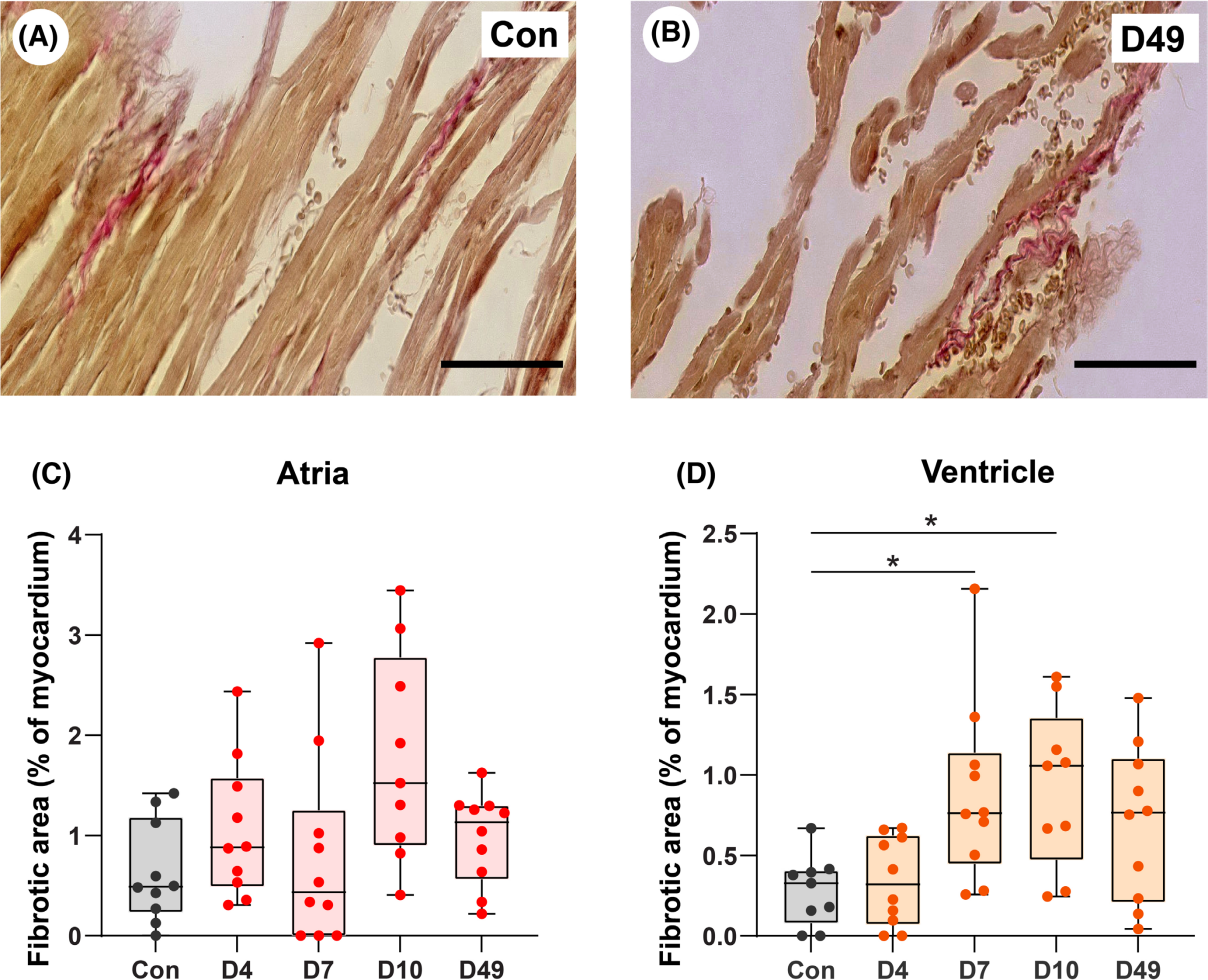
Shown are examples of fibrosis in the atrial myocardium in a (A) control (Con) and (B) viral myocarditis (VM) mouse on Day 49 (D49) after CVB3 infection. Scale bar = 50 µm. In addition, the percentages of fibrosis in the atrial (C) and ventricular (D) myocardium in control (con, *n* = 10) and VM mice (total *n* = 39) at 4, 7, 10 and 49 days (D) after CVB3 infection are shown. Each point in the graphs represents the value of one individual mouse; the bars represent mean ± SD. *means compared with the control group, *p < .05

Atrial fibrosis was quantified as a percentage of the total atrial tissue area (Figure 3A,B). We did not observe a significant difference in the fibrotic area in the atria between control mice and VM mice on days 4, 7, 10 and 49 post‐infection (Figure 3C). Moreover, we have quantified the presence of fibrosis in the ventricular tissue of the same VM mice. We found a limited but significant increase in the fibrotic area of the ventricular myocardium on days 7 and 10 (*p* <0.05) compared with the control group (Figure 3D), albeit this did not reach statistical significance on Day 49. We found comparable quantities of fibrosis (as expressed as surface area of the myocardium), in the atria and ventricles, so the transient atrial inflammation did not coincide with an increase in atrial fibrosis.

## 4 DISCUSSION

This study aimed to analyse in time, the inflammatory cell infiltration, fibrosis and viral presence in the atria of mice after mild CVB3‐induced VM. We observed a significant increase in lymphocytes, macrophages, neutrophils and mast cells in the atrial myocardium in the acute phase of VM (days 4 and 7 post‐infection), which dissipated thereafter. Moreover, we found no evidence of cardiomyocyte death or changes in fibrosis extent and only very limited CVB3 RNA in the atria on Day 4 post‐infection. These data show that mild acute VM resulted in transient inflammation in the atria, which may predispose towards the atrial arrhythmia in the acute phase.

Few studies have reported on myocarditis‐associated histological changes in the atria. Hashimoto et al showed in their study that acute CVB3‐induced myocarditis in BALB/c mice led to the formation of atrial lesions in 67% of mice, consisting of mild‐to‐moderate cardiomyocyte necrosis accompanied by inflammatory cell infiltration, from 5 up to 21 days after infection.^7^ As local CVB3 infection of the atrial myocardium was found, the authors concluded that the damage of the atrial myocardium was a direct effect of the virus. The absence of atrial cardiomyocyte necrosis in our C3H mouse model combined with the little or no atrial presence of CVB3 seems to be in line with that conclusion. However, it also highlights a difference between these two mouse models in the extent of atrial involvement. As virus type and injection titre were the same, this difference may be related either to genetic differences between the two mouse strains or to mouse gender, as in their study, Hashimoto et al used female mice and we used male mice. However, several studies have shown that male mice are more susceptible to CVB3.^12^, ^13^

A limited increase in fibrosis in the ventricles of these mice was noted, which did not reach statistical significance on Day 49 after infection. We have observed previously in these mice that CVB3‐induced cardiac infection resulted in substantial inflammation, but concomitant relatively little cardiomyocyte loss and limited replacement fibrosis in the ventricles.^11^ These characteristics of cardiac pathology in this CVB3‐induced VM mouse model conform to a relatively mild course of VM that is similar as seen in most autopsied LM patients.^9^, ^10^, ^14^ The observed lack of changes in fibrosis extent in the atria is in line with the absence of atrial damage in our model and also is similar to what we observed in the atria of LM patients.^15^

The significant increase in infiltrated lymphocytes, macrophages, neutrophils and mast cells we observed in the atria thus appears not to have been triggered by atrial tissue damage or viral presence, but rather may be related to the systemic immune response and/or the viral infection, cardiomyocyte death and inflammation in the ventricles. The kinetics of the inflammatory cell infiltration in the atria in time after infection was indeed similar to that seen for these cell types in the ventricles of these mice,^11^ although the extent of infiltration generally tended to be lower in the atria than in the ventricles. Moreover, we have shown before in humans and rats that myocardial infarction‐induced tissue damage and inflammation in the ventricles coincided with inflammation of the atria also.^8^

Although we did not analyse atrial arrhythmia or contractility in this study, increases in immune cell infiltration and fibrosis have been described repeatedly in the atria of patients with AF.^5^, ^6^ Moreover, infiltrated lymphocytes were shown to induce altered atrial contractility, including dysrhythmia and a decrease in tension, in mice with autoimmune myocarditis.^16^ Lastly, we have shown recently that CVB3‐induced VM led to transient changes in myocardial electrical conduction that were strongly associated with the ventricular cellular inflammatory infiltrate.^11^

Therefore, the results of this study show that in mild VM, a temporary increase in inflammation induced in the atria, especially in the acute phase, may facilitate the development of atrial arrhythmia and contractile dysfunction.

## ACKNOWLEDGeMENTS

None.

## CONFLICT OF INTEREST

The authors declare that they have no conflict of interest.
